# New Hospital in Rome

**Published:** 1904-12-31

**Authors:** 


### NEW HOSPITAL IN ROME.

Under the auspices of the Piccola Compagnia di Maria, a new hospital for strangers is about to be erected in Rome. In fact the foundations are already in, and the formal laying of the foundation-stone took place last week, when his Eminence Cardinal Respigli performed the ceremony.

The scheme is supported by the inhabitants of Great Britain and the Colonies, and, of course, the British residents in Rome will be represented at the ceremony. When finished the hospital will be under British protection, and as regards admission of patients, no distinction will be made as to class, nationality, or creed. All will receive equal care and attention. It is very pleasing for us to be able to record these facts. The institution will be quite a private one, and few regulations will be needed. No infectious cases will be received. As the hospital is planned this is an essential restriction, but we cannot but regret the absence of a detached block containing a few beds for infectious diseases, as it is exactly in these cases that the greatest inconvenience is felt by temporary residents in a foreign city. The hospital will be placed in charge of a certificated matron, and a staff of fully-trained, certificated nurses will be employed. It is notable that private patients may be attended by their own medical men; and in cases where the patient wishes to remain in his hotel or apartment, trained nurses will be sent to look after him. It is not stated where the nurses have been trained or will be trained, but we venture to hope that some of them will have English certificates. We believe that the Piccolo Compagnia di Maria at 45 Via Castelfidardo at present sends out nurses, and a villa for the reception of patients will continue to be used until the new hospital is opened. There is also an American hospital at 62 Via Palestra where patients are received at rates varying from 25 francs to 35 francs a day; but this has no connection with the new hospital.

The site of the new hospital is a magnificent one. It is on the Cœlian Hill not far from the Basilica of St. Stefano in Rotundo which Pope Simplicius is supposed to have converted into a church in the year 468, and standing here a fine panorama of the Sabine Hills lies before us.

The plan of the new building is that of a Latin cross. In length it is a little over 106 metres, and in width through the arms it is 76 metres. The limbs of the cross are 15 metres wide. The base of the lower limb is 25 40 metres by 7, and this part will form the out-patient department, dispensary, and various offices. The operating theatre will be on the first floor. The right arm of the cross will be occupied as the home of the sisters in charge; in the left will be the rooms for convalescents, and the upper and lower limbs will constitute the hospital proper. No detailed plans have yet reached us, but ere the hospital is finished we hope we shall be able to publish the ground and first-floor plans with our critical remarks. In the meantime it may, perhaps, be permissible to express a fear that the adoption of the cruciform plan may militate against the production of a really good hospital in all its parts and arrangements. For instance, the best aspect for a hospital ward is one which is south-east, or thereabouts, and is plainly impossible to obtain this when the plan is that of a cross, and the upper and lower limbs used for the wards. One of them must be of wrong aspect. Why, therefore, such a design should be deliberately chosen for a new hospital is to us a mystery, when there are plenty of really good plans.

When the foundations were being got out various archæological treasures were unearthed. Among others were Corinthian capitals of the second century, marble tablets and slabs with inscriptions dating back to the same period, bronze and copper coins of the reigns of Domitian and Diocletian, two large terra cotta amphoræ, and many other relics of antiquity. Near here was also the site of one of the earliest benedict monasteries, and not far off is the monastery of Gregory the Great; the Church of St. Gregory being built on the site of the old Palace.

It would seem that the Piccola Compagnia di Maria is a sort of child or offshoot of the Little Company of Mary in Sydney, Australia, which manages the Lewisham Hospital in that colony. This hospital lately celebrated its thirteenth anniversary, when an address was delivered by Dr. Kelly, the coadjutor Archbishop of Sydney, in presence of the Governor of the Colony, Sir Harry Rawson, and a large party of friends and sympathisers. We shall include a notice of the Lewisham Hospital in our serial "Glances at the Hospitals."

